# Editorial: CNS pain circuits in health and disease

**DOI:** 10.3389/fncir.2022.977404

**Published:** 2022-08-12

**Authors:** Francesco Ferrini, Peter A. Goldstein, Charalampos Labrakakis

**Affiliations:** ^1^Department of Veterinary Sciences, University of Turin, Turin, Italy; ^2^Department of Psychiatry and Neuroscience, Université Laval, Quebec City, QC, Canada; ^3^Department of Anesthesiology, Weill Cornell Medicine, New York, NY, United States; ^4^Department of Medicine, Weill Cornell Medicine, New York, NY, United States; ^5^Feil Family Brain & Mind Research Institute, Weill Cornell Medicine, New York, NY, United States; ^6^Department of Biological Applications & Technology, University of Ioannina, Ioannina, Greece; ^7^Institute of Biosciences, University Research Center of Ioannina, Ioannina, Greece

**Keywords:** circuits, chronic pain, dorsal horn and brain, microglia, mPFC, parabrachial nucleus, cannabinoids, neurotensin

Noxious somatosensory information detected by peripheral nociceptors is transmitted to the spinal cord where it is first processed within the dorsal horn (DH) neuronal circuits. The resulting refined information is then transmitted to several discrete brain regions whose collective activity will ultimately result in the conscious perception of pain. Information processing in distinct local circuits within the CNS can shape the specific experience of pain and the behavioral outcome. Consequently, plastic alterations in the function of these circuits can lead to altered pain perception, notably hypersensitivity and allodynia, which are hallmarks of pathological pain.

The first central synapse in nociceptive pathways is within the superficial laminae of the spinal DH or in the trigeminal spinal nucleus caudalis. There, primary afferent fibers form synapses with inhibitory and excitatory interneurons as well as with ascending projection neurons (Todd, 2010). Subsequent intensive research efforts have shed additional light on these circuits (Koch et al., 2018). The balance between excitation and inhibition in these circuits is of critical importance for normal sensory processing. Inhibitory interneurons in the DH serve as a “gate” that constrains how low threshold mechanical inputs can influence nociceptive pathways. Such low threshold inputs, “bleeding” over into nociceptive pathways, are responsible for the expression of mechanical allodynia during chronic pain. Gradwell et al. focus on a specific subpopulation of spinal inhibitory interneurons (i.e., those that are parvalbumin-positive; PVIN), and investigate whether their functional properties change over time. They describe an increase in excitability in PVIN neurons from older mice, which suggests a circuit-level adaptation to counterbalance increased excitability in the DH that occurs during aging. Using an optogenetic approach, they also demonstrated that synaptic strength between PVINs and other non-PVIN-positive neurons increases with age. Finally, they demonstrated that there was a shift from a mixed pattern of inhibition arising from both GABAergic and glycinergic inputs to one that was predominantly GABAergic in nature. Overall, these results demonstrate age-dependent plasticity in local inhibitory circuits in the spinal DH.

An additional level of functional plasticity in the DH derives from the modulation of synaptic circuits by peptides, hormones, and other mediators (which includes endogenous opioids and monoamines originating from descending pain modulatory pathways). In the study by Zhang et al. the modulatory role of the peptide neurotensin on GABAergic/glycinergic neurotransmission and analgesia was examined. Using a variety of approaches (immunofluorescence, *in situ* hybridization, electrophysiologic recordings from acute spinal cord slices, and *in vivo* behavioral assays), they demonstrated that neurotensin increased activity of local inhibitory neurons due to activation of NR2 receptors while *in vivo*, neurotensin had both acute analgesic and antihyperalgesic effects. Again, these results highlight the role of local spinal cord circuits in regulating nociception.

The role of non-neuronal cells in the function of spinal nociceptive circuits is often overlooked. Mounting evidence over the past several decades has established the role of microglia in altering DH function during pathological pain conditions (Inoue and Tsuda, 2018). In this context, van den Hoogen et al. review the evidence on cannabinoid signaling in microglia and how such regulation might be the basis for the development of novel therapeutics for the treatment of multiple chronic pain conditions. In the current climate where efficacious alternatives to opioid-based pain relievers are desperately needed, the information provided offers important insights into how the cannabinoid-microglial pathway might be exploited for this purpose.

After spinal processing, nociceptive information from the DH is relayed to higher brain centers by projection neurons. These ascending pathways distribute the information to several brain areas including the thalamus, the parabrachial nucleus, the periaqueductal gray, and various medullary nuclei. Shah and Barik review the evidence linking pain and itch to ascending projections to the parabrachial nucleus (PBN). Itch produces nocifensive and protective behaviors to a variety of irritants, and while it utilizes distinct neuronal circuits from those that contribute to transmission of nociceptive inputs, there seems to be a degree of overlap and sharing between pain and itch circuits, with the PBN acting as a common “hub” for these two aversive sensory states. By better understanding the commonality that exists between these conditions, it may be possible to find a single approach to relieving both.

Eventually, nociceptive information is transmitted to several key brain areas where the perception of pain occurs. These include the somatosensory cortices, the insular and cingulate cortex, the amygdala (and other parts of the limbic system), as well as the prefrontal cortex (PFC). These areas process different aspects of pain perception (i.e., sensory-discriminative, affective-motivational, cognitive), but no single cortical brain area exclusively processes information related to pain; in reality, pain perception is the result of the combined interaction between these different brain areas. The medial PFC (mPFC) processes affective aspects of pain perception and has an important role in executive functions (Ong et al., 2019). Furthermore, the mPFC has been shown to be a major top-down source for the activation of descending analgesic pathways (Huang et al., 2019). On this topic, Jefferson et al. review how thalamic, hippocampal, and amygdalar inputs to the mPFC contribute to pain perception and how alterations in these inputs can lead to mPFC deactivation and chronic pain. With greater insight into how such nociceptive information is integrated across these interconnected regions comes the possibility of identifying new and improved strategies for alleviating what is currently the intractable problem of chronic pain.

Our understanding of how nociception and pain are processed in the CNS has increased dramatically over the last several years. Notably, this improved understanding has been achieved through technological advances in mapping and dissecting the underlying neuronal circuits. The collection of articles in this Research Topic well represents the current state of the art in pain research in the CNS and encompass studies in both spinal and supraspinal mechanisms. As we gain greater insight into fundamental aspects of nociceptive processing at all levels, we ideally move that much closer to the goal of providing safe and effective care to the millions of people who live and suffer with pain on a daily basis.

## Author contributions

All authors listed have made substantial, direct, and intellectual contribution to the work and approved it for publication.

## Conflict of interest

The authors declare that the research was conducted in the absence of any commercial or financial relationships that could be construed as a potential conflict of interest. For full disclosure, however, one author (PG) is a co-inventor on patents related to the development of alkylphenols for the treatment of neuropathic pain and serves on the Scientific Advisory Board for Akelos, Inc., a research-based biotechnology company that has secured a licensing agreement for the use of those patents.

## Publisher's note

All claims expressed in this article are solely those of the authors and do not necessarily represent those of their affiliated organizations, or those of the publisher, the editors and the reviewers. Any product that may be evaluated in this article, or claim that may be made by its manufacturer, is not guaranteed or endorsed by the publisher.
